# Opus 12's CO_2_ transformation and the importance of collaboration

**DOI:** 10.1016/j.isci.2021.102677

**Published:** 2021-06-18

**Authors:** Heidi Lim

**Affiliations:** 1 Opus 12 Incorporated, 614 Bancroft Way, Berkeley, CA 94710, USA

**Figure 1.**
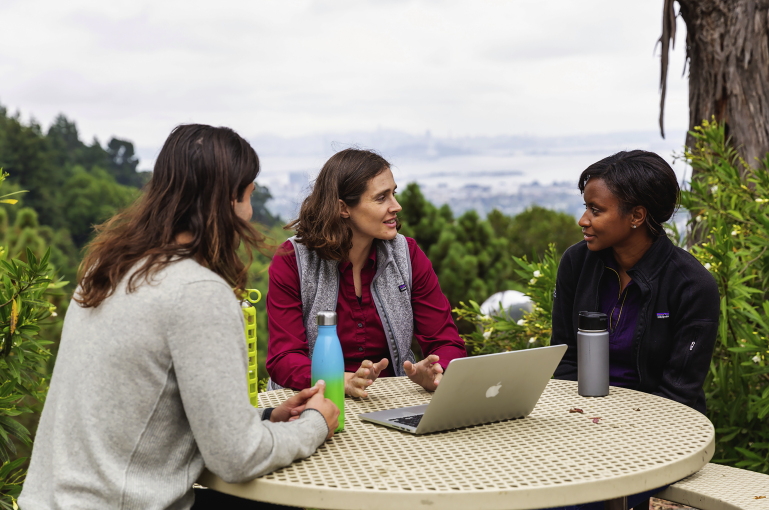
Opus 12 founding team in discussion (from left to right, Nicholas Flanders (CEO), Dr. Kendra Kuhl (CTO), Dr. Etosha Cave (CSO)). Cave is featured as an interviewee of this backstory

We can build novel systems together while leveraging their [national lab researchers] knowledge in a complementary field that, alone, we may not be able to access.

Having people take a moment to ensure that we are all on the same page is critical.

Working with researchers from adjacent fields can bring in many new ideas. The challenge sometimes is to make sure the field aligns with what we are doing.

Opus 12 is building commercial and industrial-scale carbon dioxide (CO_2_) electrolyzers to recycle CO_2_ into chemicals, materials, and fuels, which are vital to a circular carbon economy. The company was founded by three graduate students—two studied electrocatalysts within the Jaramillo Group's laboratory at Stanford and also wanted to extend their work beyond an academic setting. Cost-effective electrochemical reduction of CO_2_ is an important goal of green chemistry; CO_2_ electrolysis combines three key inputs (i.e., CO_2_, water, and electricity) and converts them into cost-competitive fuels and chemicals.

The electrolyzers mentioned above will take CO_2_ and water molecules and convert them into new molecules, such as syngas, ethylene, or methane. There are several main components to building an electrolyzer: a metal catalyst to speed up the reaction, a set of materials that surround the catalyst, and other mass transport features. Not only does Opus 12 utilize its specialized expertise and knowledge—often from a range of disciplines—to develop the electrolyzers but the company also has been leveraging universities and national laboratories to enhance in-house knowledge and technical capabilities.

According to Dr. Etosha Cave, Co-Founder and Chief Science Officer (CSO) of Opus 12 who is interviewed in this piece, teamwork and collaboration is crucial for the development of Opus 12. We also covered some of the challenges that arise when building an interdisciplinary company in this backstory.

## Proximity

### Can you share how you have worked with the broader scientific community and how you brought everyone together?

One example of a recent project is with Lawrence Livermore National Lab (LLNL). LLNL developed a set of catalysts that could be game changing for our system. These catalysts were developed in an academic setting at lower power densities, and we wanted to see if the catalysts would perform well in our reactors, which operate at higher, industrial-relevant power densities.

That project came together when someone from LLNL's upper management heard of our team and encouraged a visit in addition to a technology talk. I spoke at LLNL, sharing Opus 12's progress and challenges. From there, Opus 12 applied and was awarded a grant through the Department of Energy, which allowed LLNL and Opus 12 to work together. LLNL would send us catalyst materials to put it into our cell; we tested it and shared the results. A recent paper was published on the work in the *Journal of CO*_*2*_ *Utilization* with several members of the Opus 12 team as co-authors on that paper (Qi et al., 2021).

Furthermore, we have been working with other national labs, such as groups at the National Renewable Energy Laboratory (NREL), where they have researched an adjacent technology for many years and have expertise in some of the specific components that we also use for our system. The national labs have an abundance of knowledgeable people who are interested in supporting new technology development—especially at scale. We can build novel systems together while leveraging their knowledge in a complementary field that, alone, we may not be able to access.

## Language

### Did you encounter any challenges or any benefits from working with people of different backgrounds and expertise?

A major benefit of working together is that we can broaden our view of possible solutions. When you are working on technology in the early stages, you are so focused on the key components, but there might be other elements outside of your initial purview that could also aid in the development. Hence, having other people all work together on the core technology can move you from point A to point B much faster.

As for challenges, yes there were some. For instance, the acronym “MEA” means “membrane electrode assembly” at Opus 12. However, we late realized that MEA has a completely different meaning for other organizations. That is, MEA is “methylethylamine” for those who work in carbon capture. Therefore, there were moments in which we had to pause and clarify the definition. In the LLNL case, its catalysts were built for an academic reactor, so we had to consider how to transfer them into our industrial-scale reactor, which required further discussions and careful planning. Having people take a moment to ensure that we are all on the same page is critical.

## Research methods

### How did the decision of branching out from your core fields come about and what implication did it have on your careers?

We do recognize that this is technology is very challenging. Thus, as stated previously, bringing all ideas on the table would help us reach our goals faster. National labs already have a structure in place to work with companies (through the Cooperative Research and Development Agreements or CRADAs), so the technology transfer segment in the licensing is already more streamlined than it is with other entities. And, why not work with them? If we can get to scale faster with grant funding, this seems like a no brainer.

Moreover, joining forces with national labs opened up opportunities for additional funding; there are some requirements in these funding opportunities to work with collaborators, and the national labs have high credibility and high visibility. There are also funding avenues in which the national lab receives money in support of working on a project with a small business.

### What's the benefit to the national labs to collaborate with, like Opus 12 or commercial companies?

The national labs' main mission is to develop innovation for the country. Consequently, for them to work with a company that is commercializing a technology–of which they have worked on in the past—is a huge win-win. This helps the national labs further develop the technology, and the company can license that technology if it is useful for the product. Having a new product reach commercialization creates jobs and builds up the economy. That is the national labs' main mission.

### When publishing this or any interdisciplinary paper, how do you decide which community or venue to target and, also, what are the challenges you have faced in publishing?

Publishing the paper was definitely a team effort. As a company, we are sometimes challenged when we are building out an IP portfolio (and so are the national labs) because there are sensitivities around timing and making sure that provisional patents are submitted to the patent office before you submit for publication.

Publications seem to be more welcoming of interdisciplinary research these days—or at least there are specific ones out there that are. Broadly speaking, we do have to be sensitive to how we present our results and which results are presented. Certainly, there is a stronger push to publish since we are working with the national labs. We are writing more papers than had we not worked with the national labs. Nonetheless, it is beneficial to the company to still be connected to the academic community, and one way to stay connected is through publications.

### What's the benefit of staying connected to the academic community for a commercial company?

One strong benefit is that it helps with hiring. Often, technical talent wants to be around other scientific people. So having a national lab joint publication with a company shows that we are still doing very interesting science. I would say that is probably the greatest value. Secondarily, it breeds new potential collaborations and new possibilities for working with other researchers.

## Final thoughts

### What did you learn about interdisciplinary research from the project and what tips would you give to anyone considering undertaking such work?

Working with researchers from adjacent fields can bring in many new ideas. The challenge sometimes is to make sure the field aligns with what we are doing. If it is too far outside of the scope, then it can be too much to try to blend the fields together. Having both parties that are interested and willing and wanting to work together is crucial.

There is always room for improvement. Nevertheless, researchers have managed to establish a streamlined contract; there is a standard and clear way of managing the IP that might come out of the contract, and there is a licensing office that is available to discuss licensing agreements. These are nice incentives to have, and I would encourage more of them in a more streamlined pathway.

### Do you have any other thoughts or advice for the *iScience* community or areas where you see this type of collaboration continuing to grow?

Having access to a multi-user facility such as the Molecular Foundry at Lawrence Berkeley National Laboratory has been super helpful for us. We use characterization and diagnostic equipment that is specialized and expensive. It has been super helpful to not have to purchase these multimillion dollar instruments and, instead, use those funds to hire researchers. These benefits have streamlined and accelerated our technology development in many ways.

**Figure 2:**
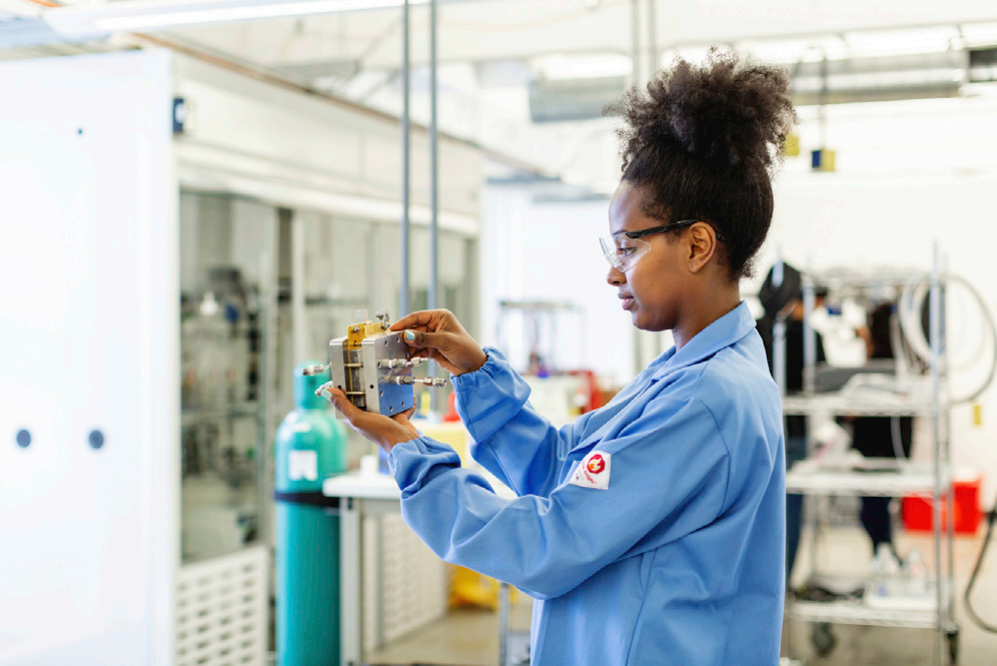
Opus 12 team member holds a CO2 electrolysis test cell

**Figure 3:**
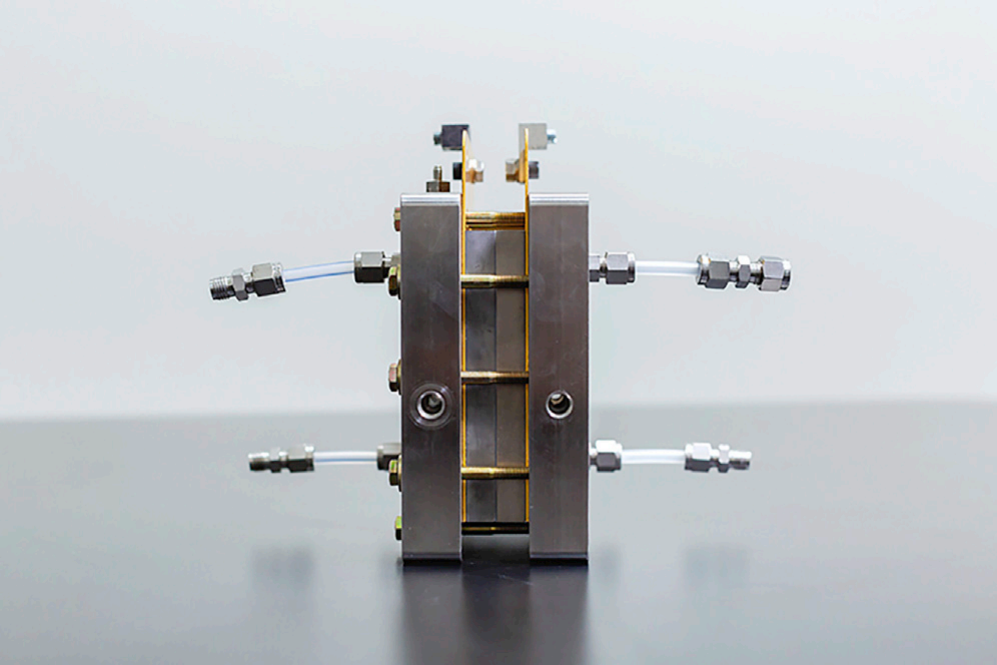
An Opus 12 CO2 electrolysis test cell
